# Coupling of Thalamocortical Sleep Oscillations Are Important for Memory Consolidation in Humans

**DOI:** 10.1371/journal.pone.0144720

**Published:** 2015-12-15

**Authors:** Mohammad Niknazar, Giri P. Krishnan, Maxim Bazhenov, Sara C. Mednick

**Affiliations:** 1 Department of Cell Biology & Neuroscience, University of California Riverside, 900 University Ave, Riverside, CA, 92521, United States of America; 2 Department of Psychology, University of California Riverside, 900 University Ave, Riverside, CA, 92521, United States of America; University of Oxford, UNITED KINGDOM

## Abstract

Sleep, specifically non-rapid eye movement (NREM) sleep, is thought to play a critical role in the consolidation of recent memories. Two main oscillatory activities observed during NREM, cortical slow oscillations (SO, 0.5–1.0Hz) and thalamic spindles (12–15Hz), have been shown to independently correlate with memory improvement. Yet, it is not known how these thalamocortical events interact, or the significance of this interaction, during the consolidation process. Here, we found that systemic administration of the GABAergic drug (zolpidem) increased both the phase-amplitude coupling between SO and spindles, and verbal memory improvement in humans. These results suggest that thalamic spindles that occur during transitions to the cortical SO Up state are optimal for memory consolidation. Our study predicts that the timely interactions between cortical and thalamic events during consolidation, contribute to memory improvement and is mediated by the level of inhibitory neurotransmission.

## Introduction

New memories need to be transformed into more stable representations or they will be forgotten [1]. It is well established that sleep is a period optimized for this process of consolidation [2], [3]. Systems consolidation, the coordination of memory-related brain regions of hippocampus, neocortex, and thalamus, is thought to occur during NREM sleep [4]. This sleep phase is characterized by thalamically-driven oscillatory activity called sleep spindles (12–15 Hz) and large amplitude, synchronized cortically-driven slow oscillations (SOs). Early NREM sleep (i.e., Stage 2) is characterized by a large number of spindles and sporadic SOs. During late (Stage 3/4) NREM sleep, SOs are the dominant electrophysiological event consisting of Up and Down states [5] that are hypothesized to orchestrate cortical neural activity by synchronously silencing during the Down states and firing during the Up states. Coordinated spindles and SOs have been reported in several brain areas, including thalamus, neocortex [5], amygdala, and striatum [6] in later NREM sleep [7]. However, the extent of the temporal coordination between these major sleep rhythms during Stage 2 NREM sleep has not been established.

Spindles and SOs have independent features that correlate with improvements in hippocampal-dependent memory formation [8], [9]. Neuromodulation of sleep via pharmacology or transcranial current stimulation has shown that increasing the number of spindles [8] or SOs [10] in a targeted manner increases declarative memory, suggesting an independent causal role for each electroencephalographic (EEG) event. Further, previous studies have observed a temporal coupling of spindles and SOs during NREM sleep [2], which increases following a memory task [11]. These findings suggest that consolidation may benefit from appropriate coordination of these EEG features. The functional consequence of spindle and SO event coordination during Stage 2 sleep, however, is not known.

To address this question, we analyzed human EEG to determine the importance of a precise temporal relationship between SOs and spindles during NREM Stage 2 sleep for hippocampal-dependent, declarative verbal memory performance. We defined spindle/SO complexes as epochs with SOs whose amplitude exceeded a set threshold and preceded a spindle (see Methods). The properties of these complexes and the specific phase-amplitude relationships between them were correlated with performance changes after a nap. Specifically, we examined the coupling between the phase of the SOs and amplitude of the spindles following a verbal memory task. Phase-amplitude coupling has been shown across various frequencies and brain regions [12], [13]. Further, hippocampal gamma-theta coupling has been shown to be critical for working memory and other cognitive tasks [14]. Here, we investigated whether cross-frequency coupling of spindles and SOs can be linked to increased memory consolidation. We tested this possibility using a pharmacological manipulation (i.e., zolpidem (Ambien)) that increased sleep spindles and improved declarative verbal memory over placebo, during a daytime nap [8]. In addition, we used sodium oxybate (Xyrem) as a comparison hypnotic, which decreased spindle activity [15]. We then compared the contributions of each electrophysiological event independently and coupled using regression models.

## Materials and Methods

### Participants

Data were collected and previously reported upon by Mednick and colleagues [8]. A total of 28 participants (14 female) between the ages of 18–39 (22± 3 years) who were normal sleepers and habitually obtained approximately 8 hours of sleep each night signed written informed consent to participate in the experiment, which was approved by the Institutional Review Board of the University of California, San Diego.

### Study Design

Each participant was tested in a repeated-measures, crossover design in which each participant experienced three drug conditions (10mg of zolpidem, placebo and 2.5mg of sodium oxybate). Drug condition order was randomized. Each condition was separated by 5–10 days to allow for drug wash-out and recovery from any sleep changes related to the nap and/or study drugs. Zolpidem is a GABA agonist and binds to GABA-A α1 subunit of the GABA receptor. Binding of zolpidem in the GABA receptor results in opening of Cl- leading to hyperpolarization of the neuron [16], [17]. GABA-A is widely expressed across the brain regions in cortex, thalamus and brainstem regions. Sodium oxybate is metabolized to gamma-hydroxybutric (GHB) acid, which is a GABA-B agonist and also binds to G-protein coupled receptors for specific for GHB [18].

After a night of sleep with polysomnography (PSG)-monitoring, participants were woken at 5AM and given breakfast. At 6AM, participants were tested on a paired associates verbal memory task. At 8AM, subjects went back to their bed for an electrode check and nap. Drugs were administered immediately before lights out. This timing was chosen as the best method for establishing a uniform measure of sleep latency. The nap took place at 8:30AM to capitalize on circadian fluctuations in REM sleep (highest in the morning). This maximized differences in sleep stages between the drug and placebo conditions with higher levels of REM sleep in the placebo condition, and greater number of sleep spindles in the zolpidem condition. Subjects were allowed to sleep for up to two hours of time in bed or until they achieved 90min of sleep. Sleep was scored on-line to ensure that all subjects had the same total sleep time [8]. Subjects were continuously monitored during the hours between the nap and the second test session (3PM). They were allowed to watch TV, eat lunch, shower, read and work on the lab computer.

### Sleep Data Analyses

**Fig 1:**
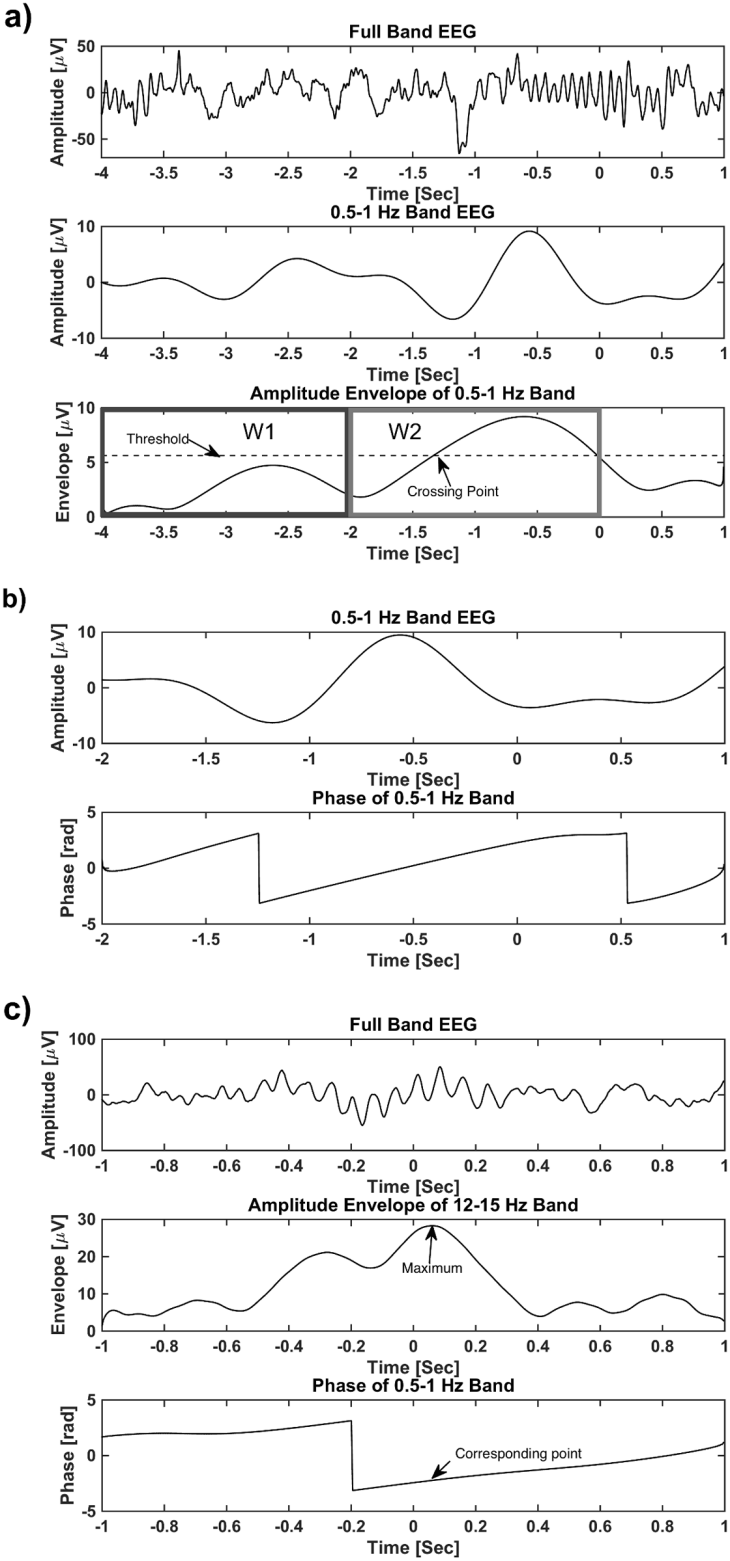
(**a**): Identification of spindle/SO complexes. Mean, μ, and standard deviation, σ, of the amplitude envelope of slow oscillations is calculated via the Hilbert transform in the reference window (W1) to build a threshold as T = μ +2*σ. Then the amplitude envelope of slow oscillations was measured in a pre-spindle window (W2) to determine the presence of above-threshold slow oscillations that occurred prior to the spindle. (**b**): Phase of a sample signal in 0.5–1 Hz frequency band. (**c**): Phase of slow oscillations, provided by Hilbert transform, at the maximum of the spindle amplitude envelope.

Trained polysomnographic technicians visually identified and placed markers on spindles in the EEG records. The initial markers, based on visual detection, were not places at specific positions (e.g. spindle center). In order to have a consistent location of the marker within the spindle, each recording was first filtered to spindle frequency band (12–15 Hz), then the data within four-second windows centered at the visually detected markers were considered. Amplitude envelopes of these four-second windowed signals were calculated. The windowed data that had the highest peak envelope was considered as the template, as it had the strongest spindle. Finally, the cross correlation function between the template and the other windows was calculated and the markers were shifted based on the peak position of the cross correlation function. EEG segments in Stage 2 sleep from the left central scalp electrode (C3 based on the International 10–20 system) containing spindles (based on the spindle marker) and SO were detected using a threshold method. Given that prior literature has shown that one of the locations for highest spindle power in the central channels [19], [20], and that the number of spindles was significantly higher on C3 compared to C4 for all the conditions (zolpidem: paired t-test p = 5.1082e-04, placebo: paired t-test p = 2.830e-04, sodium oxybate: paired t-test p = 0.008) in this dataset, we processed data from C3. We examined stage 2 sleep, since spindle could be identified by visual inspection. Further, both slow oscillation and spindles are known to occur during stage 2 sleep [21]. The data were then filtered to the 0.5–1 Hz frequency band resulting in s_0.5–1_ (Fig 1a). Next, a reference window (W1, between 2–4 seconds prior to spindle marker) was used to estimate the baseline amplitude of each SO. Then, a pre-spindle window (W2 between 0–2 seconds prior to spindle marker) measured the presence of above-threshold SO that occurred prior to the spindle. Mean, μ, and standard deviation, σ, of the amplitude envelope of s_0.5–1_ was calculated via the Hilbert transform in the reference window (W1) to build a threshold as T = μ +k*σ. Here, k = 2 was chosen, since it performed best on detecting SO at visual inspection. This threshold was used to detect a spindle/SO complex, which was counted when the amplitude envelope of s_0.5–1_ exceeded the threshold in W2. Only EEG segments consisting of spindles and SO were used for further analysis.

Power within the spindle window (one second before and one second after the spindle marker) was computed using fast Fourier transform (FFT). Phase of slow oscillations was calculated based on the Hilbert transform of s_0.5–1_ signal (Fig 1b). Then, the value of this phase at the peak of spindle amplitude envelope was calculated in each spindle window (Fig 1c).

Modulation index is a measure to determine the degree of coupling between the amplitude of a faster rhythm and the phase of a slower rhythm. To calculate this index, *M*, we first constructed a composite complex-valued signal by combining the amplitude of the faster signal, $a_{s_{12-15}}$, with the phase of the slower signal,$\;\phi_{s_{0.5-1}}$, provided by their Hilbert transforms:

$$z\left[n\right]=a_{s_{12-15}}\left[n\right]\text{exp}\left(i\;\phi_{s_{0.5-1}}\;\left[n\right]\right)$$ (1)

The normalized mean of this composite vector across trials relates to how the coupling between phase and amplitude vary across trials. This is given formally by the following (Canolty et al, 2006):

$$M_{raw}=\left\lceil \frac{1}{N}\sum_{n=1}^{N}z[n]\right\rceil$$ (2)

where |.| denotes the absolute value. In this way, *M*_*raw*_ is a measure of the degree of asymmetry of the probability density function of *M*_*raw*_. If the distribution of $\phi_{s_{0.5-1}}$ is uniform, any departure of the distribution of *z*[*n*] from radial symmetry will indicate $a_{s_{12-15}}$ and $\phi_{s_{0.5-1}}$ share mutual information and $a_{s_{12-15}}$ is dependent on $\phi_{s_{0.5-1}}$. Therefore, a non-zero value of *M*_*raw*_ can be a useful measure of phase-amplitude coupling [22]. However, the mean *M*_*raw*_ must first be normalized before it can be used as the degree of coupling between $a_{s_{12-15}}$ and $\phi_{s_{0.5-1}}$, rather than the statistical properties of either $a_{s_{12-15}}$ or $\phi_{s_{0.5-1}}$ examined alone [13]. This can be done by generating a distribution using a surrogate data approach by introducing an arbitrary time lag between $\phi_{s_{0.5-1}}$ and $a_{s_{12-15}}$. Our implementation of the normalized modulation index is based on the MATLAB code provided in the supplementary material of [13]. We calculated this measure within the window of spindle/SO complex (two seconds before and one second after the spindle marker).

### Paired Associates Memory Task

In the word pair associates task, subjects were visually presented 48 semantically related word pairs. Study words were two-syllable English words between four and seven letters in length and of moderate frequency (e.g. table-bench). Research assistants rated words on a 1–7 imagery scale as having imagery ratings between 5–7. Word pairs (with the second word presented under the first to avoid lateralization effects) were presented on a computer screen placed at a distance of about 50 cm from the eyes. The letters were black on a white background and centered. Paired-associate lists of 144 words are arranged in 6 groups of 24 pairs, which were randomized across conditions. Subjects were presented with two word-pair lists in the morning session. Recall was cued, i.e., subjects were presented with the first word of each pair and had to recall the second one. Immediate recall was tested immediately after training, and subjects were shown the correct answer after each response. Delayed recall was tested during the post-intervention testing session. Memory consolidation was measured as a differences score between the number of words recalled in the delayed recall test relative to the immediate recall test.

## Results

As previously reported, verbal memory performance and sleep spindle density (spindles/Stage 2 minutes) were significantly higher in zolpidem compared with placebo (paired t-tests for performance: p = .036, and for spindle density: 3.447±1.399 vs. 2.864±1.789, p = .002), spindle density decreased in sodium oxybate (1.965±1.317, p = .001), and no performance differences were found between sodium oxybate and placebo (p = .210), although performance was numerically lower in the sodium oxybate condition [8]. We first examined each sleep event (SO and spindle) within the spindle window individually and their relationship with memory performance. We found no difference in power of spindle and SO between placebo, zolpidem and sodium oxybate conditions (repeated measures p = .141 and p = .224 for spindles and SOs, respectively). Next, we combined the data from all the conditions and assessed correlations between the power in spindle and SO within the spindle window and performance improvement. Although this analysis that combines all conditions and treats subjects as independent samples has a significant pitfall because it ignores the repeated measures nature of the data, we performed it as an exploratory overall analysis and reported the results. Both spindle power and SO power was correlated with performance improvement when data from all groups were combined (spindle power: r = .320; p = 0.003; SO power: r = .223; p = 0.040). We also performed this correlation for each condition separately. The spindle power was correlated with performance improvement in zolpidem (r = .450; p = .016) and sodium oxybate (r = .454; p = .015) conditions, but not in placebo (r = .184; p = .348). Furthermore, SO power was correlated with performance improvement in placebo (r = .384; p = .043), but neither in zolpidem (r = .100; p = .612) nor sodium oxybate (r = .229; p = .240). Statistical tests were then conducted to examine if the correlation coefficients were significantly different between conditions for spindle and SO power based on Fisher’s transformation. The correlation between spindle power and performance improvement were not significantly different between different drug conditions (zolpidem vs. placebo p = .289, sodium oxybate vs. placebo p = .284, and zolpidem vs. sodium oxybate p = .984). Additionally, correlations between SO power and performance improvement did not differ between the drug conditions (zolpidem vs. placebo p = .280, sodium oxybate vs. placebo p = .638, and zolpidem vs. sodium oxybate p = .541). These results suggest that the association between power of SO or spindle with performance improvement was similar across different drug conditions. We next investigated whether the temporal coordination between thalamic spindles and cortical SOs was a stronger indicator of memory improvement.

**Fig 2:**
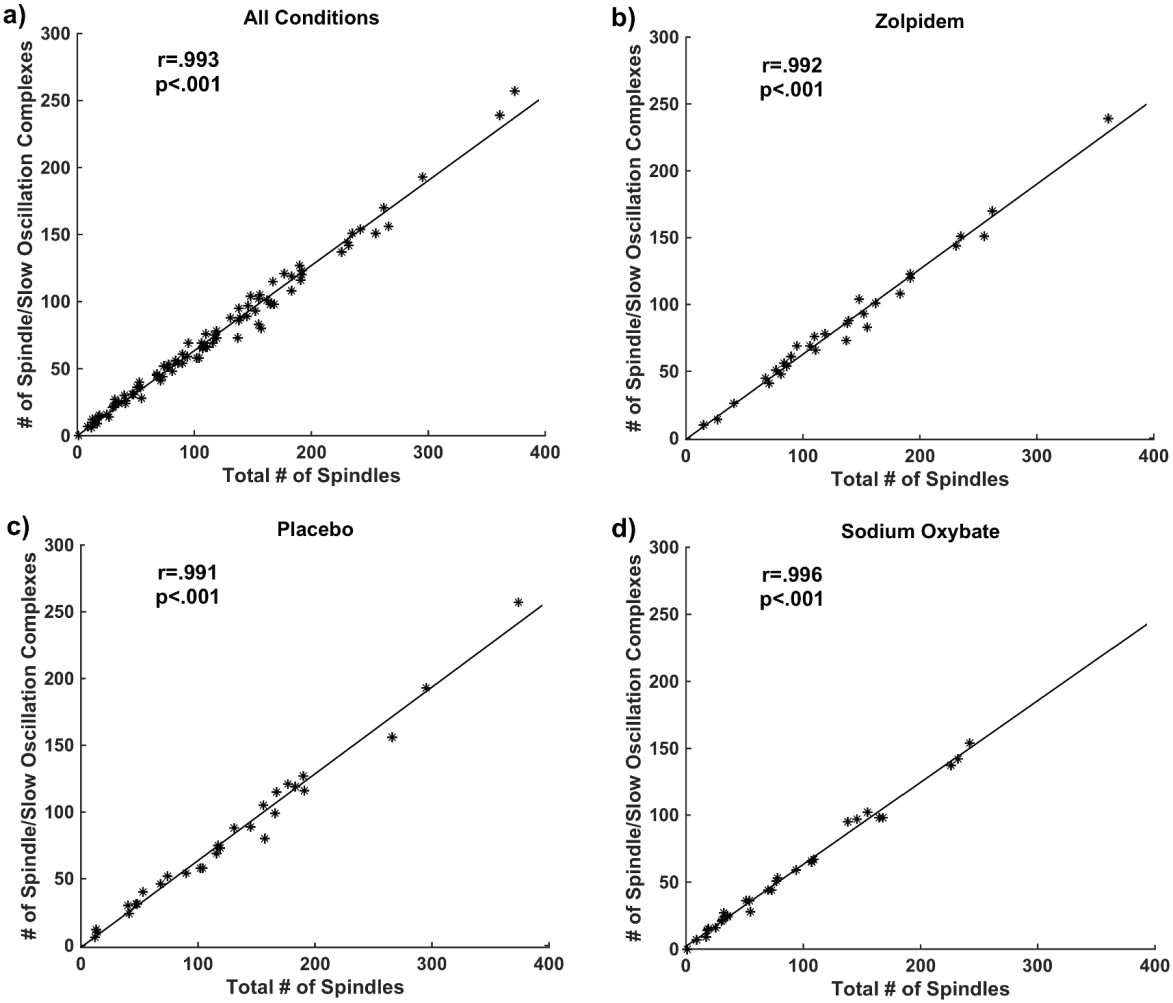
Spindles and Spindle/SO Complexes: During Stage 2 sleep, the number of spindle/SO complexes and the number of spindles were highly correlated in all subjects suggesting a functional relationship. **(a)**: All conditions, **(b):** zolpidem, **(c):** placebo **(d):** sodium oxybate.

We first investigated the relationship between spindles and SOs by simply examining the correlation between the number of spindles and the number of spindle/SO complexes (numbers of spindles and complexes and comparison between different drug conditions are reported in Tables 1 and 2, respectively) for the combined conditions (r = .993 and p < .001), placebo (r = .991 and p < .001), zolpidem (r = .996 and p < .001) and sodium oxybate (r = .992 and p < .001) (Fig 2a–2d). Because there were differences in number of spindles across conditions, we checked whether these were also differences in the proportion of spindles to spindle/SO complexes and found no significant difference of across the three conditions (p = .773). This indicates that the number of spindles is almost a perfect predictor of the number of spindle/SO complexes in each condition regardless of spindle number. As this result is based on a local thresholding approach to identify SOs, we also applied a global thresholding method in which an EEG amplitude threshold was considered for detection of slow oscillations [21]. The results were still significant: for 40 mV: combined conditions (r = .844 and p < .001), zolpidem (r = .787; p < .001), placebo (r = .884; p < .001), sodium oxybate (r = .872; p < .001), and for 60 mV: combined conditions (r = .616 and p < .001), zolpidem (r = .557; p = 0.001), placebo (r = .789; p < .001), sodium oxybate (r = .598; p < .001). Nevertheless, we used local thresholding that provides a larger number of complexes for a more inclusive analysis. We hypothesize that this close relationship between spindles and spindle/SO complexes may not be accidental, but rather may have a functional significance.

**Table 1:** Numbers of spindles and spindle/SO complexes across different drug conditions.

	Number of spindles	Number of complexes
Zolpidem	4123	2598
Placebo	3704	2365
Sodium oxybate	2599	1652

**Table 2:** t-test pair comparison between drug conditions on number of spindles and spindle/SO complexes.

	Number of spindles	Number of complexes
Placebo vs. zolpidem	p = .192	p = .280
Placebo vs. sodium oxybate	p = .009	p = .007
Zolpidem vs. sodium oxybate	p = 5.291e-04	p = 6.848e-04

Repeated-measures ANOVA on numbers of spindles: p<001.

Repeated-measures ANOVA on numbers of spindle/SO complexes: p<001.

**Fig 3:**
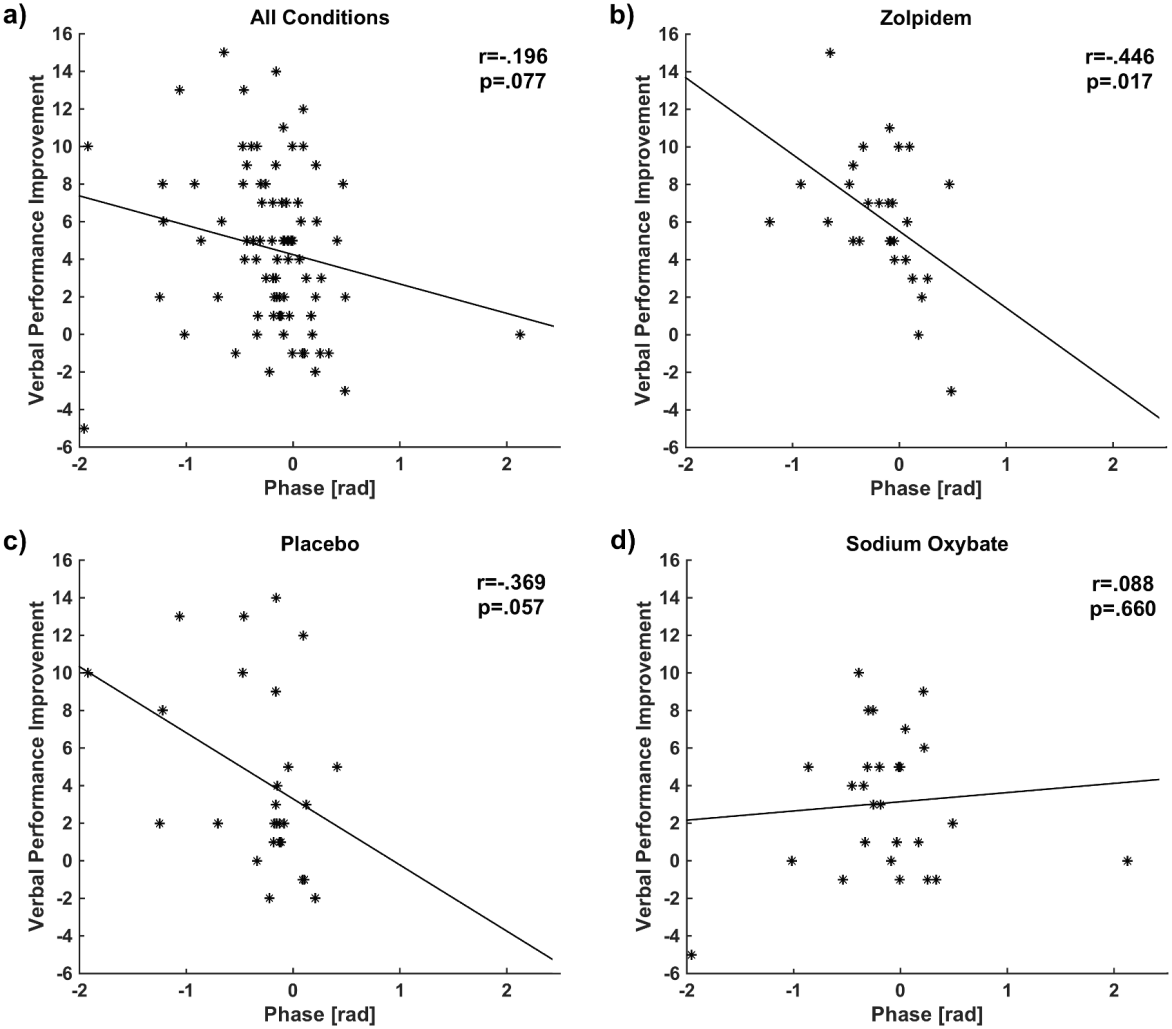
Memory performance improvement and phase-amplitude timing: SO phase at spindle peak was correlated with memory improvement in zolpidem and placebo conditions suggesting a general mechanism of memory formation. This relationship was not observed in sodium oxybate, which showed the worst performance. Phase value of each subject corresponds to the average value over all spindle/SO complexes in that subject. **(a)**: All conditions, **(b):** zolpidem, **(c):** placebo **(d):** sodium oxybate.

Next we investigated the phase-amplitude timing of the spindle/SO complex. For this analysis, in each spindle window, the instantaneous SO phase at the peak spindle amplitude envelope was calculated for each condition. Zero phase of SO ($\phi_{s_{0.5-1}}\;=\;0$ = 0) corresponds to the approximate positive peak of the oscillation (Fig 1b). We found that the phase values of SO at the peak of spindle envelope had less deviation in the zolpidem condition (μ = -0.162± 0.389, 95% CI: -0.314 to -0.012) compared to placebo (μ = -0.305± 0.516, 95% CI: -0.510 to -0.101) and sodium oxybate (μ = -0.136± 0.663, 95% CI: -0.399 to 0.125) conditions. We then correlated these phase values with performance improvement in the combined conditions and each condition separately. Although a trend is observed for the combined conditions in Fig 3a, the correlation was not significant (r = -.196; p = .077). Nevertheless, If the three points (having phase values more than 2 or less than -1.5) far from the data center (greater than mean+3*SD, or smaller than mean-3*SD) are removed, the correlation becomes significant (r = -.267; p = .017). Moreover, a significant relationship was found in the zolpidem condition (r = -.446; p = .017, Fig 3b), and a marginal relationship was also found in the placebo condition (r = -.369; p = .057, Fig 3c), but no relationship was found in the sodium oxybate condition (r = .088; p = .660, Fig 3d). These correlations were not significantly different between zolpidem vs. placebo (p = .741). Nevertheless, the association strength in the sodium oxybate was significantly different from the zolpidem condition (p = .044) and revealed a trend when compared to the placebo condition (p = .093). These findings also indicate a “preferred phase” in SO for the occurrence of spindle events that predicted better memory consolidation in both zolpidem and placebo, but this effect was not seen in the comparison hypnotic. The last was likely because a relatively small number of spindles in the sodium oxybate condition led to performance improvements insufficient to detect the effect of coordination of SO and spindles on memory improvement. In zolpidem and placebo, the optimal SO phase for a spindle to occur that is correlated with improvement in memory performance was during the rising of the SO, or the beginning of the Up state, indicated by negative correlations in Fig 3a and 3b [5], [7].

**Fig 4:**
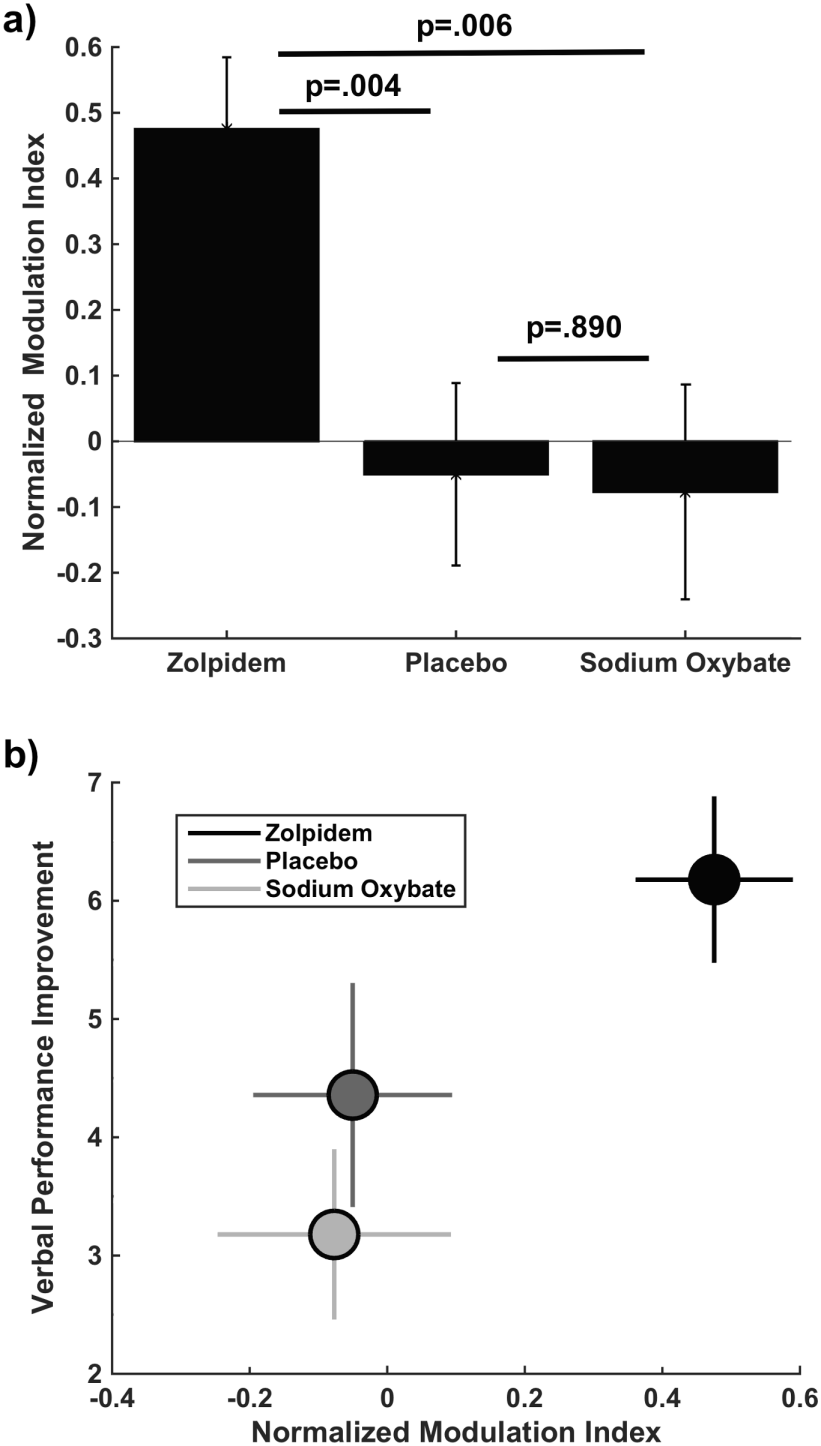
**(a):** Normalized modulation index for spindle/SO complexes. **(b):** Normalized modulation index and memory performance improvement: Modulation Index in each drug condition (zolpidem (black circle), placebo (dark grey), sodium oxybate (light grey) are shown relative to change scores in verbal memory.

To compare the strength of coupling between SO and spindle events within the spindle/SO complex, we computed the normalized modulation index for each drug condition, which measures the reliability of the temporal relationship between the phase of SOs to the amplitude of spindles (see Methods). Higher index values indicate that spindle amplitude peaks consistently at a certain phase of the SO, while lower values indicate higher variability in this relationship. The normalized modulation index for spindle/SO complexes was significantly higher with zolpidem compared to placebo and sodium oxybate conditions (repeated measures ANOVA: p = .005, paired t-test: zolpidem vs. placebo p = .004, sodium oxybate vs. placebo p = .890, Fig 4a). This calculation of the relationship between spindles and SOs did not correlate with performance improvement (zolpidem: r = .053; p = .787, placebo: r = -.196; p = .316, sodium oxybate: r = .199; p = .308). This suggests that phase of the slow oscillation at the time moment of maximal spindle power is more accurate predictor of verbal performance improvement than modulation index (modulation index measures phase relationship for the entire window). The findings from phase analysis and modulation index taken together suggests that there is a greater degree of temporal coordination of thalamic (spindle) and cortical (SO) oscillations under the condition where verbal memory is improved (i.e., zolpidem) (Fig 4b).

Our analysis of the phase of SO at the peak of spindle envelope suggests that thalamocortical coordination is strongly related to performance improvement. At the same time, performance improvement was inconsistently correlated with spindle and SO power individually. Since we found that spindle events are so tightly coupled with spindle/SO complexes, it is difficult to tease apart the contribution to memory consolidation of each electrophysiological event individually (i.e., spindle and SO power) versus the coupled event (i.e, SO phase at peak of spindle). For this purpose we employed regressions in order to measure the non-confounded or independent contribution of each of the components to memory. For the combined conditions and each drug condition separately, we tested the contribution of spindle power, SO power and the SO phase at spindle peak on verbal memory improvement (Table 3). The role of these parameters would differ across the conditions since different drugs have different impact on spindles and slow oscillations. The regression was significant for the combined conditions, (p < .001), explaining 19% of the variance, with both SO phase at spindle peak (p = .002) and spindle power (p = .010) as significant predictors, but not SO power (p = .142). Similarly, the regression was significant for zolpidem, (p = .007), explaining 31% of the variance, with both SO phase at spindle peak (p = .016) and spindle power (p = .025) as significant predictors, but not SO power (p = .996). The regression was also significant in placebo (p = .017), explaining slightly less variance than zolpidem (R^2^ = .267), and only SO phase at spindle peak was a significant factor (p = .015), compared with spindle power (p = .581) and SO power (p = .103). For sodium oxybate, the model was not significant (p = .079) and explained the least amount of variance (R^2^ = .153), and only spindle power was a significant predictor of memory improvement (p = .022), compared with SO power (p = .728) and SO phase at spindle peak (p = .848). Thus, our analysis revealed that the condition that showed the best performance (zolpidem) had the most reliable (i.e., lowest variance) phase-amplitude coupling, as measured by modulation index, and both SO phase at spindle peak and spindle power contributed to performance. For the two conditions with lower performance, the modulation index was low and performance improvement was inconsistently supported by sleep events. Placebo had low modulation index, but its regression was still significant and SO phase at spindle peak was a predictor. Finally, the sodium oxybate condition that showed decreased spindle activity, also had low modulation index values, and the lowest regression significance, with only spindle power as a predictor.

**Table 3:** Regression results for the combined conditions and each condition separately.

	SO power	Spindle power	Phase	Significance	Variance
Combined conditions	p = .142	p = .010	p = .002	p < .001	19%
Zolpidem	p = .996	p = .025	p = .016	p = .007	31%
Placebo	p = .103	p = .581	p = .015	p = .017	26%
Sodium oxybate	p = .728	p = .022	p = .848	p = .079	15%

## Discussion

To summarize, we found several corroborating indicators that memory consolidation is influenced by both the number of spindles and the precise temporal coordination of spindles with SOs. First, spindle peak timing during the rising phase of SO predicted performance improvement in the best (zolpidem) and middle performing (placebo) conditions. This phenomenon was also observed when we combined all conditions and treats subjects as independent samples. We pharmacologically enhanced this effect with zolpidem, which also increased the modulation index and spindle density [8]. Spindles and spindle/SO complexes were tightly coupled in all subjects, consistent with the idea that increasing the number of spindles in NREM Stage 2 increased the number of spindle/SO complexes. Additionally, more of these spindles at the preferred SO phase (i.e., the transition from the Down state to the Up state of SO) contributed to greater memory performance in the zolpidem group, compared with placebo. In contrast, sodium oxybate, with numerically the lowest memory performance, decreased spindle activity, reduced the modulation index, and did not show any consistent relationship between spindle/SO complexes and memory performance. Altogether these results suggests that thalamocortical events must be tightly coordinated to favor memory formation, and that the coordination of the oscillatory events may be a better indicator of consolidation than individual EEG events. Importantly, the same phase-amplitude relationship was shown in both zolpidem and placebo groups and also for the combined conditions, indicating that this finding may predict a general principle of sleep-dependent memory that occurs naturally, and not a pharmacologic artifact.

Animal studies demonstrated that spindle oscillations arise from inhibitory interaction within the thalamic network [23]. GABAergic synaptic projections within reticular thalamic neurons and between reticular neurons to thalamic relay cells leads to recurrent inhibitory induced oscillations at the spindle frequency [24], [25], [26]. Zolpidem, a GABA-A agonist, may increase spindle oscillations by enhancing the inhibitory neurotransmission in the thalamus. Further, GABA-mediated inhibitory activity balances excitatory activity during the Up-states of SO [27] and influences the duration and termination of Up-states [28]. Computational models predicted that higher inhibition due to elevated activity of GABAergic interneuron leads to increased synchronization of SO [29]. Together these studies suggest that zolpidem may lead to increase in spindles and synchrony with SO and can facilitate temporal binding of these rhythms during Stage 2 sleep, a period in which these events are normally less coordinated than during late (Stage 3/4) NREM sleep, since spindles are known to occur in distributed fashion compared to SO [30]. Coupling between these oscillations during the Up state in Slow Wave Sleep (SWS) is expected. However, during Stage 2 sleep specifically, spindles and SO are less synchronized and therefore their timing and the impact of that timing on consolidation are unknown. Thus, we examined cross frequency coupling between SO and spindles during Stage 2 sleep and its effect on memory improvement.

Results from our study on the relationship between cross frequency coupling and memory performance during sleep is consistent with rodent studies demonstrating that different stages of memory processing (e.g., encoding, consolidation and retrieval) are preferentially active during distinct phases of cortical theta oscillations marked by higher gamma oscillation [31], [32]. Since spindles are associated with massive increases in intracellular Ca^2+^ [33], which is required to induce long-term potentiation, a coincidence of thalamic spindles with other sleep EEG events, such as hippocampal sharp waves [34] at the Down to Up transition phase of cortical SO may be necessary to form permanent memories. Consistent with this hypothesis, impaired anteroposterior propagation of cortical slow waves is associated with mistiming of thalamic spindles and hippocampal ripples in the schizophrenia mouse model [35], which may reflect sleep-dependent memory consolidation failures in patients with schizophrenia [36].

Precise thalamocortical interaction has been hypothesized to be important for human memory consolidation [2]. Studies in humans have shown a temporal relationship between SO and spindles [21], that strengthened after learning. In addition, enhancing SO by transcranial stimulation increased both memory performance and spindles [10]. Taken together, these earlier studies suggest a functional link between the two sleep rhythms. Here, we present the first evidence that memory performance in humans benefits from the occurrence of spindles during the transition to the Up state of the SO during Stage 2 sleep, indicating a functional importance of precise electrophysiological event coordination during sleep in defining mechanisms of memory consolidation.
